# Conventional and diffusion-weighted magnetic resonance imaging findings of benign fibromatous paratesticular tumor: a case report

**DOI:** 10.1186/1752-1947-5-169

**Published:** 2011-05-03

**Authors:** Athina C Tsili, Maria I Argyropoulou, Dimitrios Giannakis, Nikolaos Sofikitis, Konstantine Tsampoulas

**Affiliations:** 1 Department of Clinical Radiology, University Hospital of Ioannina, Leoforos S Niarchou, 45500, Ioannina, Greece; 2 Department of Urology, University Hospital of Ioannina, Leoforos S Niarchou, 45500, Ioannina, Greece

## Abstract

**Introduction:**

The vast majority of paratesticular masses are benign. Magnetic resonance imaging of the scrotum may provide valuable information in the pre-operative work-up of scrotal masses, by allowing the precise localization of the lesion and helping in characterizing its nature. Diffusion-weighted magnetic resonance imaging is an evolving technique that can be used to improve tissue characterization, when interpreted with the findings of conventional magnetic resonance sequences. We present the case of an adenomatoid tumor of the tunica albuginea, with abundant fibrosis evaluated by magnetic resonance imaging of the scrotum, including both conventional and diffusion-weighted sequences. To the best of our knowledge, there are very few reports in the English literature regarding the magnetic resonance imaging features of this rare benign paratesticular tumor and no report on the diffusion-weighted magnetic resonance findings. We discuss the value of magnetic resonance imaging in the pre-operative diagnosis of benign fibromatous paratesticular tumors and differential diagnosis.

**Case presentation:**

A 45-year-old Caucasian man was referred to us with a palpable left scrotal mass. Magnetic resonance imaging of his scrotum revealed the presence of a multilobular left paratesticular mass, mainly detected with very low signal intensity on T2-weighted images and restricted diffusion on apparent diffusion coefficient maps. These findings were suggestive of a fibrous component, and were confirmed on histology following lesion excision.

**Conclusion:**

Magnetic resonance imaging of the scrotum, by using both conventional and diffusion-weighted sequences, could have a potential role in the evaluation of scrotal masses.

## Introduction

Determining the accurate location of a scrotal mass, whether intratesticular or paratesticular is extremely important pre-operatively, to ensure adequate treatment planning. Most paratesticular masses are benign, therefore radical orchiectomy may be obviated [1,2]. Magnetic resonance imaging (MRI) of the scrotum may represent a useful diagnostic tool for the morphologic assessment and tissue characterization in the pre-surgical work-up of scrotal masses [1,2].

Adenomatoid tumors are benign mesothelial neoplasms, accounting for approximately 30% of all paratesticular neoplasms [1-4]. The majority (77%) of these tumors arise from the epididymis. They may also arise from the testicular tunica (14%) and, less often, from the spermatic cord and the testicular parenchyma [1-4]. We present a case of an adenomatoid tumor of the tunica albuginea, with abundant fibrotic component, evaluated by conventional and diffusion MRI.

## Case presentation

A 45-year-old Caucasian man presented to our Urology department with a palpable left scrotal mass, known for two years, which had progressively enlarged during the last three months. He reported no history of epididymitis, torsion or trauma. On clinical examination the mass was painless, firm and mobile. His serum tumor markers, including alpha-fetoprotein, beta-human chorionic gonadotropin and lactate dehydrogenase, were normal.

**Figure 1:**
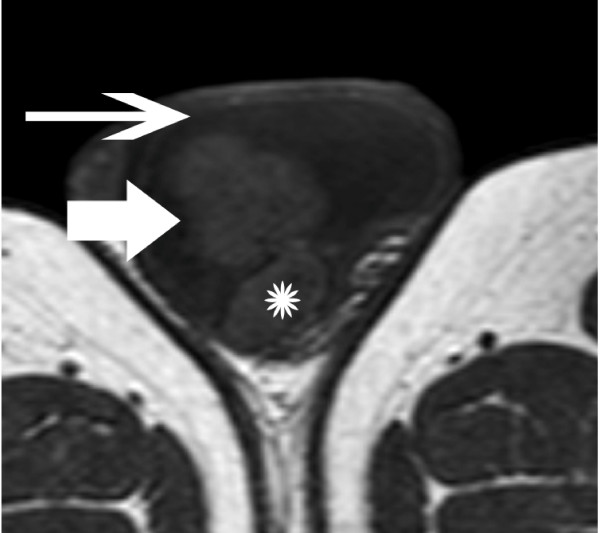
**Transverse T1-weighted image depicts a multilobular left scrotal mass (arrow), located in the paratesticular space**. The lesion had similar signal intensity, when compared to the normal testicular parenchyma (asterisk). Left hydrocele (long arrow).

**Figure 2:**
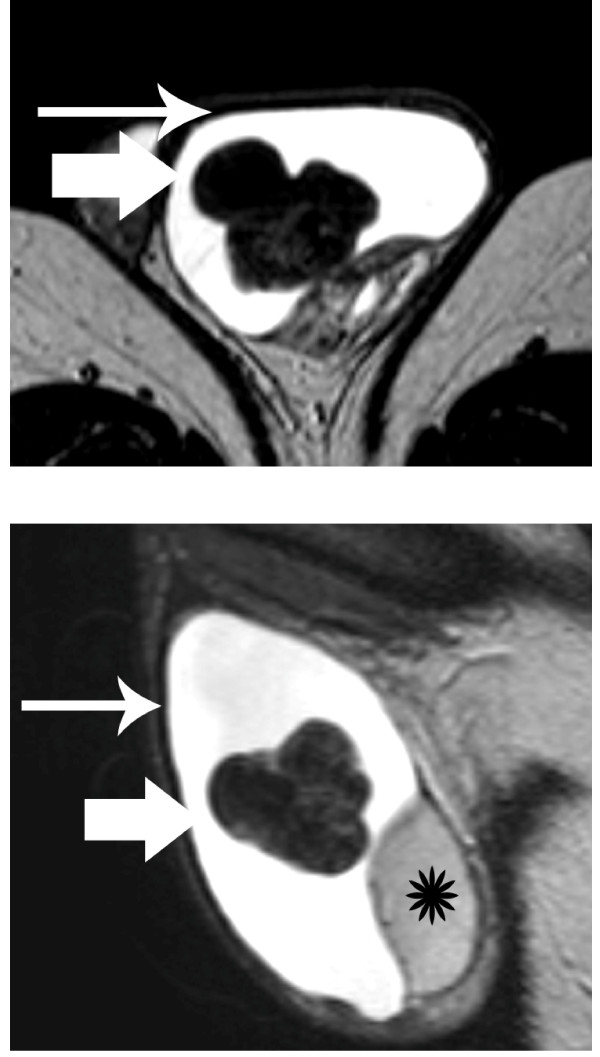
**T2-weighted images (a) Transverse and (b) sagittal T2-weighted images show tumor heterogeneity**. The mass (arrow) was mainly hypointense on T2-weighted images, a finding suggestive of the presence of fibrous tissue. Left hydrocele (long arrow). Normal left testis (asterisk).

**Figure 3:**
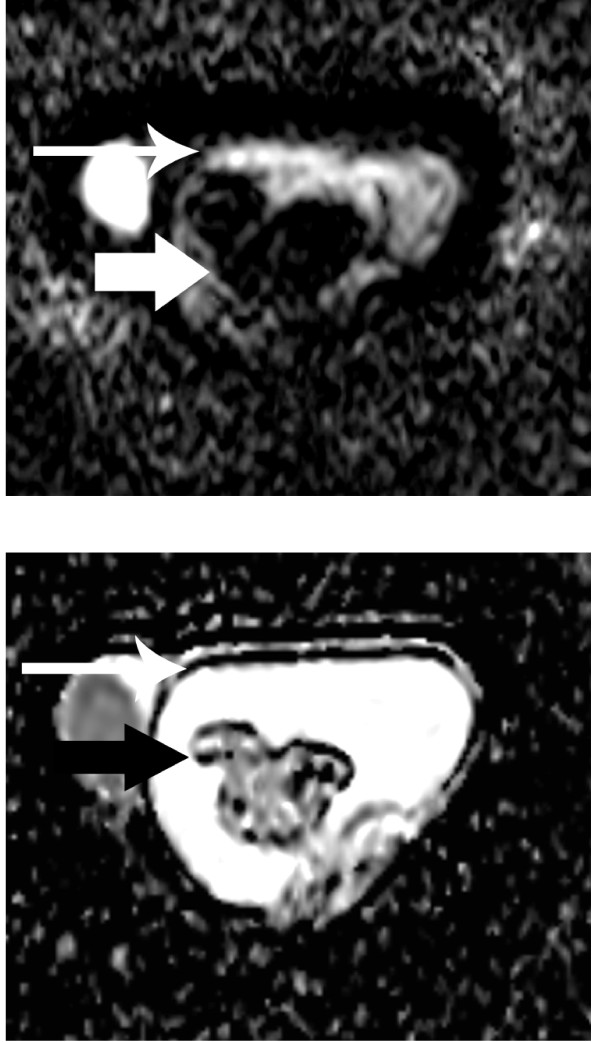
**(a) Transverse DW echo planar image (*b *= 900 mm^2^/s) and the (b) corresponding ADC map**. The mass (arrow) appears mainly hypointense on DW images, due to the presence of abundant fibrous part. The ADC value of the fibrous component was 0.86 × 10^-3^mm^2^/s. Left hydrocele (long arrow).

Sonographic examination showed a sharply-demarcated hypoechoic, vascular left paratesticular mass, located close to the head of his epididymis. A large left hydrocele, with low level echoes was also found. MRI evaluation of the scrotum was done on a 1.5-T magnet unit, using a pelvic phased-array coil. The study included fast spin-echo axial, sagittal and coronal T2-weighted sequences and spin-echo axial T1-weighted sequences. Diffusion imaging was performed in the axial plane, using a single shot, multi-slice spin-echo planar diffusion pulse sequence. The maximum *b*-value was 900 s/mm^2^. A multilobular left paratesticular mass (Figures 1, 2, 3), in close proximity to the testicular tunicae of the superoanterior aspect of his left testis was detected. The dimensions of the tumor were 33 × 34 × 32 mm. T1-weighted images demonstrated a mass isointense to his testicular parenchyma (Figure 1). The mass was heterogeneous on T2-weighted and apparent diffusion coefficient (ADC) maps, with areas of high T2 signal and ADC value of 1.56 × 10^-3^mm^2^/s, and others of very low T2 signal and ADC value of 0.86 × 10^-3 ^mm^2^/s (Figures 2a, b, 3b). A large, left hydrocele, with a few septa and ADC value of 2.93 × 10^-3^mm^2^/s was also revealed. Both of his testicles, his epididymis and his spermatic cords were normal. The mean ADC value of his testicular parenchyma was 0.94 × 10^-3 ^mm^2^/s and that of the epididymis 1.37 × 10^-3^mm^2^/s. His left testicular tunicae were intact. Based on MRI findings, the diagnosis of a benign fibromatous paratesticular tumor was suggested. Therefore, our patient underwent local excision of the mass. Histopathology reported an adenomatoid tumor of the tunica albuginea, with abundant fibrosis. Our patient is now well, without signs of disease on clinical and sonographic examination, one year after surgery.

## Discussion

Solid neoplasms of the paratesticular tissues are rare [1,2]. They affect patients of all ages, most commonly presenting as a slow-growing non-tender scrotal mass, as it was in our case. Adenomatoid tumors are the most common epididymal neoplasms, followed by leiomyomata [1-4]. These tumors are usually unilateral, more often seen on the left side, as in our patient. They are benign neoplasms; no cases of malignant transformation or local recurrence have been reported [3,4].

MRI of the scrotum as an alternative imaging modality has been proven with satisfactory results in the evaluation of scrotal contents [1,2,5-7]. The wide field-of-view, multiplanar capability and high resolution of this technique allow the precise demonstration and lesion localization, thereby distinguishing paratesticular from intratesticular mass lesions [1,2,5-7]. Moreover, tissue signal intensity may prove valuable in characterizing their nature [1,2,5-7].

MR examination of the scrotum in our patient revealed the presence of a sharply-demarcated multilobular paratesticular mass, isointense on T1-weighted images, heterogeneous, but predominantly of very low signal intensity on T2-weighted images. One limitation of the MR protocol used in this study was that it did not include post-contrast images, although the lesion was reported with vascularity on sonographic examination. The hypointensity of the mass on T2-weighted images and the restricted diffusion on ADC maps was suggestive for the presence of fibrous tissue, proved through histology to correspond to the abundant fibrotic component of an adenomatoid tumor of the tunica albuginea. The presence of abundant collagen-producing fibroblastic cells and a dense network of collagen fibres cause restriction in the diffusion of the water molecules in fibrotic lesions, as also proved in our patient [8,9]. Patel *et al*. reported a case of an adenomatoid tumor of the tunica albuginea evaluated by MRI [3]. The tumor was also of low signal intensity on T2-weighted images, with decreased enhancement after gadolinium administration, when compared to that of normal testicular parenchyma in our report [3].

Differential diagnosis of benign fibromatous paratesticular masses, as in our case, should include fibrous pseudotumor. This rare tumor is not a true neoplasm, but a reactive fibrous proliferation of the extratesticular tissues [1,2,10,11]. The majority (75%) of cases arise from the tunica vaginalis, and the remaining from the epididymis, the spermatic cord and the tunica albuginea [1,2,10,11]. MRI findings include signal hypointensity on both T1 and T2-weighted images, a finding strongly suggesting the fibrous nature of the mass. After gadolinium administration, little or no enhancement of the tumor has been reported [1,2,10,11].

## Conclusion

MRI evaluation in our patient provided valuable information in the pre-operative work-up, by allowing the precise localization of the mass and helping in characterizing the benign nature of fibrous paratesticular tumor, by using both the conventional and diffusion MRI. Confirmation of the diagnostic efficacy of MRI examination with prospective studies in unselected scrotal masses is required.

## Abbreviations

ADC: apparent diffusion coefficient; DW: diffusion-weighted; MRI: magnetic resonance imaging.

## Consent

Written informed consent was obtained from the patient for publication of this case report and any accompanying images. A copy of the written consent is available for review by the Editor-in-Chief of this journal.

## Competing interests

The authors declare that they have no competing interests.

## Authors' contributions

ACT, MIA and KT were major contributors in writing the manuscript. PG and NS had contribution to conception and data acquisition, and also in writing this manuscript. All authors read and approved the final manuscript.
